# Effects of different kinds of anti-Alzheimer’s disease drugs on cognitive improvement: protocol for a systematic review and network meta-analysis of phase III clinical trials

**DOI:** 10.1186/s13643-022-01964-x

**Published:** 2022-05-03

**Authors:** Diyang Lyu, Min Gong, Yong Zhang, Xuanxin Lyu

**Affiliations:** 1 Capital Medical University, Beijing, 100069 China; 2 Dongzhimen Hospital, Beijing University of Chinese Medicine, Beijing, 100700 China; 3 Neurological Rehabilitation Center, Beijing Rehabilitation Hospital, Capital Medical University, Beijing, 100144 China

**Keywords:** Alzheimer’s disease, Drug therapy, Systematic review, Network meta-analysis, Phase III clinical trial, Randomized controlled trial

## Abstract

**Background:**

Alzheimer’s disease is a neurodegenerative disease characterized by progressive cognitive decline and dysfunction of independent living ability, with huge economic and healthy burden worldwide. However, there is still a lack of effective long-term drugs to improve cognitive function and reduce or halt disease progression. Phase III clinical trials of anti-AD drugs based on different hypotheses were in the pipeline, and this protocol for a systematic review and meta-analysis aims to determine what is the most effective direction for the development of drugs on cognitive improvement.

**Methods/design:**

We will search the following literature databases for eligible studies from inception to December 2021: Ovid MEDLINE, Ovid Embase, PubMed MEDLINE, and Cochrane Central Register of Controlled Trials. Google Scholar, *ClinicalTrials.gov* registration platform, and the AlzForum website will also be searched for additional studies. Studies will be included irrespective of publication status or language. Phase III clinical trials reporting on the effect of anti-AD drugs on participants with AD will be included. Two independent reviewers will screen the hit articles and identify phase III clinical trials, extract data, and assess the quality of each study individually. The Cochrane Risk of Bias tool 2 (RoB 2) will be used to assess the risk of bias. For each kind of drugs based on the corresponding hypothesis, we will compare the study design and demographic features of the clinical trials and include appropriate studies in the network meta-analysis. The primary outcomes will be the indicators of cognitive improvement. The secondary outcomes will be activities of daily living, neuroimaging changes, biomarkers, and safety. Through network meta-analysis, we will suggest the hypothesis that most likely to improve cognitive function and provide the ranks of all kinds of drugs. We will give recommendation grade of each comparison using the Confidence In Network Meta-Analysis (CINeMa) tool.

**Discussion:**

This study will provide helpful evidence for further drug development and clinical practice for treating Alzheimer’s disease.

**Systematic review registration:**

PROSPERO CRD42021251507

**Supplementary Information:**

The online version contains supplementary material available at 10.1186/s13643-022-01964-x.

## Background

The population with dementia has increased over the last few decades. It was reported that there were 50 million people in 2018, and this population was estimated to triple in 2050 worldwide [1]. Alzheimer’s disease (AD), characterized by progressive cognitive decline and dysfunction of independent living ability, is the most common cause of dementia [2]. With the aging of the world population, AD will cause more and more huge years of life lost (YLL) [3]. The global economic burden of AD was heavy and has been predicted to rise from $1 trillion in 2018 to double in 2030 [4]. These conditions markedly affect the quality of life of older individuals and constitute a major public health problem. Hence, this poses a worldwide severe challenge to the prevention and treatment of AD.

AD is a multifactorial and heterogeneous neurodegenerative disease. The understanding of the pathological mechanisms of AD has gone through a long process. Alois Alzheimer first reported this disease in 1906 [5]. Scientists presented the neurofibrillary tangles through electron microscopy in 1963 [6], isolated the amyloid-β (Aβ) in 1984 [7], and revealed the core pathological changes of AD. In the recent decades, various hypotheses were put forward regarding the cause of AD, including Aβ hypothesis [8], tau hyperphosphorylation hypothesis [9], cholinergic hypothesis [10], mitochondrial cascade hypothesis [11], calcium homeostasis hypothesis [12], inflammatory hypothesis [13], neurovascular hypothesis [14], metal ion hypothesis [15], and lymphatic system hypothesis [16]. Based on the above hypotheses, scientists have developed a large number of drug candidates, hundreds of which have been validated for their efficacy in cognitive protection through clinical trials [17].

Up to 2019, there were 2173 clinical trials on various hypotheses of AD [17]. Among them, the Aβ hypothesis was the most heavily tested, followed by the neurotransmitter hypothesis, the mitochondrial cascade and related hypotheses, and the tau propagation hypothesis. Since tacrine was approved by Food and Drug Administration in 1995 [18], five drugs have been approved by Food and Drug Administration which were all developed based on the neurotransmitter hypothesis, including one *N*-methyl-*D*-aspartic acid (NMDA) receptor antagonist (memantine) and four cholinesterase inhibitors (donepezil, galantamine, rivastigmine, and tacrine). However, scientists found that the above-mentioned drugs could only slow the symptomatic progression with limited clinical benefit [19, 20]. Besides, recent systematic reviews provide new evidences that anti-Aβ therapy [21, 22], anti-inflammation therapy [23], regulation of lipid metabolism [24], etc. were unable to prevent cognitive decline with obvious benefits or reverse the underlying pathology continuously.

Due to the poor efficacy of therapeutic drugs based on classical hypotheses, the number of the drugs based on other hypotheses has increased gradually in recent 5 years [25]. There were 13 anti-Aβ trials among the total 24 ongoing phase III clinical trials in 2016 [26], and 6 among 29 in 2020 [25]. Accordingly, phase III clinical trials based on different hypotheses, including tau, synaptic plasticity/neuroprotection, metabolism/bioenergetics, inflammation/infection/immunity, neurovascular, neurogenesis/growth factor/hormone, epigenetic, proteostasis/proteinopathies, and symptomatic based hypotheses, were in the pipeline [25]. In addition, the number of repurposed agents was increasing, comprising 43% of the pipeline in 2020 [25]. The growing number of drugs based on new hypotheses and repurposed agents suggests that the therapeutics of AD may in a new stage. Scientists are trying to develop anti-AD agents through more directions. However, the efficacy and safety of these drugs have not been compared directly or indirectly with each other.

A drug clinical trial is often time-consuming, requiring the recruitment of tens to thousands of subjects at a cost of billions of dollars. It may be helpful for further clinical practice and drug development to analyze existing clinical trials and determine the potential key hypotheses associated with cognitive prevention or to reverse disease progression. As a result of the long treatment course, routine administration and dosage, large sample size with randomized controlled study design and multiple outcome measurements, phase III clinical trials can comprehensively evaluate the therapeutic efficacy and safety of the drugs for patients with specific disease, comparing with those phase I/II clinical trials. Thus, we will include phase III clinical trials to investigate the difference of benefits among drugs developed from different hypotheses of AD.

Network meta-analysis (NMA) is an approach to synthesize data across more than two comparisons and to provide results from both direct and indirect comparisons [27]. We aim to perform a systematic review and NMA to investigate the rank of efficacy among different kinds of anti-AD drugs. To our knowledge, this will be the first such study. By constructing a network, drugs developed based on the same hypothesis will be grouped into one node respectively, and the thickness of each edge connected the nodes will represent the count of related phase III trials. In this way, the network will display all the existing direct comparisons and provide further indirect comparisons among different kinds of drugs. As phase III clinical trials usually compare the experimental group with the placebo group, there may be only indirect treatment comparisons. The results will be the pooled estimation for each comparison between drugs of interest to determine the cognitive benefits of different kinds of drugs. In summary, we will perform a systematic review and network meta-analysis of phase III clinical trials to compare the effect and safety of drugs based on different hypotheses and placebo to provide a better choice for the treatment of patients with AD. Through this study, we will reveal the potential key hypothesis which is most likely to affect the clinical efficacy, and provide evidence for further clinical practice and new drug development.

## Methods

### Study guidelines and registration

This protocol for the systematic review and network meta-analysis follows the guidelines of the Preferred Reporting Items for Systematic Review and Meta-Analysis (PRISMA) Protocols (PRISMA-P) statement [28] (Additional file 1). The study will be conducted from November 30, 2021, to June 30, 2022, following the Cochrane Handbook [29], and reported following the PRISMA statement [30] and its extension for NMA [31]. This protocol is registered in the International Prospective Register of Systematic Reviews (PROSPERO, 251507 submitted). Any amendment will be clarified as supplementary material in the further systematic review.

### Data source and search strategy

The following electronic literature databases will be searched using our search strategy from inception to December 2021: (i) Ovid MEDLINE; (ii) Ovid Embase; (iii) PubMed MEDLINE; (iv) Cochrane Central Register of Controlled Trials (CENTRAL). We will also search *ClinicalTrials.gov* registration platform and Google Scholar for missing studies and ongoing clinical trials. In addition, the AlzForum website (https://www.alzforum.org/) will also be searched as its “THERAPEUTICS” database comprehensively summarizes AD-related drugs and corresponding clinical trials. We will also check the reference list of each hit literature which enters the full-text screening step or each review article in this field. No language restriction will be applied to the articles. The search strategy is presented in Table 1 and supplementary material.

**Table 1:** Search strategy for PubMed MEDLINE

Search	Query
#1	alzheimer disease [MeSH Terms]
#2	Dementia [MeSH Terms]
#3	(((alzheimer*[Title/Abstract]) OR (“alzheimer disease”[Title/Abstract])) OR (“alzheimer’s disease”[Title/Abstract])) OR (dement*[Title/Abstract])
#4	controlled clinical trials, randomized [MeSH Terms]
#5	(((((((randomized controlled trial) OR (controlled clinical trial)) OR (randomi?ed[Title/Abstract])) OR (placebo[Title/Abstract])) OR (Drug Therapy[MeSH Subheading])) OR (random*[Title/Abstract])) OR (trial[Title/Abstract])) OR (groups[Title/Abstract])
#6	Humans [MeSH Terms]
#7	((phase III[Title/Abstract]) OR (phase 3[Title/Abstract])) OR (phase III[Title/Abstract])
#8	#1 OR #2 OR #3
#9	#4 OR #5
#10	#9 AND #6
#11	#8 AND #7 AND #10

### Eligibility criteria

The eligibility criteria for this systematic review and network meta-analysis are presented in Table 2.

**Table 2:** Eligibility criteria using the PICOS (participants, intervention, control, outcome, study design) format

Item	Content
Participants	Patients with AD
Intervention	Anti-AD drugs
Control	Placebo
Outcomes	Clinical outcomes: cognitive improvements (e.g., Alzheimer’s Disease Assessment Scale-Cognitive section), activity of daily living (Alzheimer’s Disease Cooperative Study ADL scale).
	Biomarker outcomes: plasma and cerebrospinal fluid biomarkers (e.g., amyloid-β, tau)
	Neuroimaging outcomes: structural magnetic resonance imaging (e.g., whole brain volume, hippocampal volume), positron emission computed tomography (e.g., standard uptake value ratio of glucose, amyloid-β, tau), etc.
	Safety outcomes: adverse events, serious adverse events, death, etc.
Study design	Phase III clinical trials, which are always randomized controlled trials

### Participants

We will include the studies that recruited patients with AD. The diagnosis should be based on clearly reported diagnostic criteria, including the NINCDS-ADRDA criteria [32], the IWG-2 criteria [33], or the “ATN” framework [34]. We will exclude the patients with other subtypes of dementia, with mild cognitive impairment (or prodromal AD) or subjective cognitive decline (or preclinical AD) in this study. We will not place restrictions on the demographic indicators for the participants of original clinical trials. Gender, age, education level, human race, combined medication, etc. will not be restricted in this systematic review as reported clearly.

### Interventions and comparators

The intervention in the experimental groups should be anti-AD drugs only. Non-drug therapy will be regarded as transcranial magnetic stimulation, diet therapy, physical exercise, cognitive stimulation, single or multi-domain intervention, etc. We will not limit the dosage form, the way of administration, or the dosage. However, included drugs should be considered to be associated with a clear pathogenic hypothesis. Comparing with experimental groups, control groups should receive placebo to achieve consistency.

### Outcome measurements

The primary outcome will be a cognitive improvement. The related indicators including Alzheimer’s Disease Assessment Scale-Cognitive section (ADAS-Cog) [35], Clinical Dementia Rating-Sum of boxes (CDR-SOB) [36], and Mini-Mental State Examination (MMSE) [37] will be regarded as primary outcomes. The activities of daily living (ADL), biomarkers (Aβ and tau, etc.), neuroimaging (magnetic resonance imaging or positron emission tomography), and safety indexes (adverse events, death, etc.) will be included as secondary outcomes. We will not restrict the duration of the study and the time point of the evaluation for each outcome.

### Study design

We will only include randomized controlled trials (RCTs). As we mentioned above, only phase III clinical trials will be included into our systematic review. Units of randomization could be individuals or clusters. Quasi-RCTs or any other study design will not be regarded as RCT. The extension studies will usually be excluded for mismatch baseline treatment, but open-label studies will be considered.

### Study selection

**Fig. 1:**
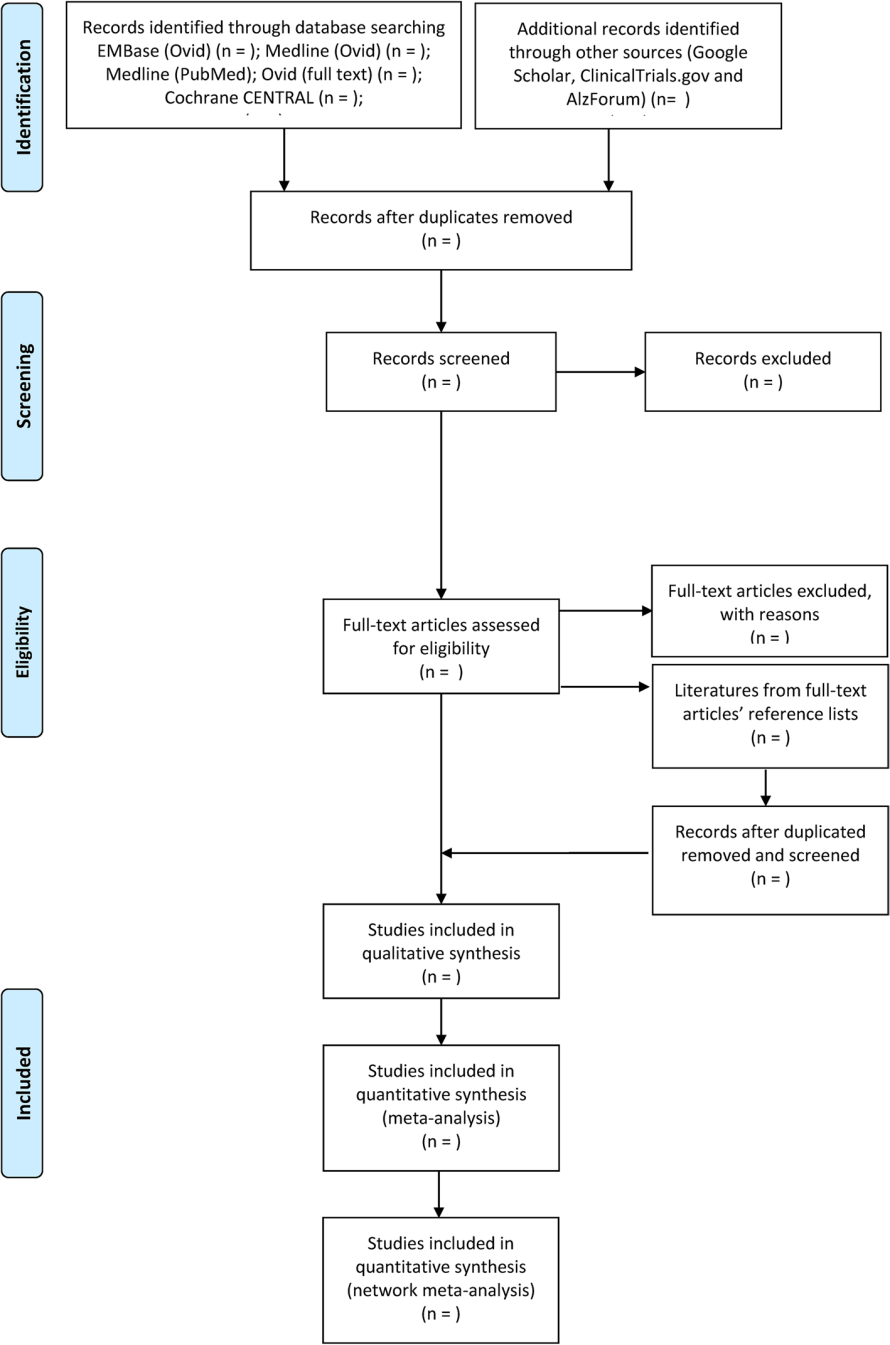
The PRISMA flow diagram of this systematic review and network meta-analysis

In this step, we will remove duplicated articles with the EndNote X9 software firstly. Then, two independent reviews (DL and MG) will review all titles and abstracts of hit articles, and exclude those studies unrelated with our systematic review obviously following the established criteria individually. After that, the reviewers will evaluate the full text of the existing articles, further exclude the studies that do not meet the criteria, and record the reasons one by one. A third reviewer (XL) will deal with any disagreement between the two reviewers when necessary. Any disagreement will be recorded with detailed reason by the reviewers. When finished, the rate of agreement will be calculated and reported in the further systematic review. Details of the selection procedure will be presented as a PRISMA flow diagram (Fig. 1).

### Data extraction

Data will be independently extracted with an established template by using Microsoft Excel software. Two reviewers (DL and MG) will extract data individually. After data extraction, the consistency between the two reviewers will be compared, any discrepancy will be resolved by the third reviewer (XL). Required information includes demographic data, stage of AD, combined medication at baseline, diagnostic criteria and inclusion/exclusion criteria, primary and secondary outcome measurements, and the usage of the drugs. We will also extract methodological information for the next step “quality assessment”. Results from original clinical trials will be extracted as mean ± standard deviation (SD) with sample size for continuous variables, and number with percentage for categorical variables. Any missing data or information will be obtained through sending an e-mail to the corresponding authors when necessary, including those unpublished manuscripts, conference abstracts, and the registered studies that have reached the completion date. A detailed schedule of data and information to be extracted is listed in Table 3.

**Table 3:** Data and information extraction schedule

Subject	Content
Publication information	First author and correspondence author, e-mail, publish year, country, corporate sponsorship, percentage of authors from sponsoring company
Participant	Recruitment source, sample size, age, gender, human race, diagnostic criteria, stage of AD, family history, inclusion and exclusion criteria
Intervention	Drug name, based hypothesis, administration, dosage and usage, frequency of the treatment and the treatment course
Control	Placebo, administration, dosage and usage, frequency of the treatment and the treatment course
Outcome	Outcome measurements and each assessment time point, cognitive or biomarker endpoints, adverse events, and the detailed data
Study design	Study duration, treatment and follow-up course, study sites, the application of randomization and blinding, description about statistical analysis, sample size calculation
Other information	Attendance rate, reasons for withdrawing, combined treatment of AD

### Quality assessment

We will apply the Cochrane Risk of Bias tool 2 (RoB2) [38] to assess the methodological quality for each included randomized controlled trial. RoB2 tool presents five domains of risk of bias, including the bias arising from the randomization process, bias due to deviations from intended interventions, bias due to missing data, bias in the measurement of the outcome, and bias in the selection of the reported result. Bias of each domain is calculated from several methodological issues, and the tool will help provide an “overall risk of bias” for each randomized trial based on the five domains [38]. Two independent reviewers (DL and MG) will follow the guidelines of the RoB2 tool [38] and Cochrane Handbook [29] to assess the following fields of each RCT: randomization and allocation, blinding, outcome evaluation and statistical analysis, outcome reporting. For missing details, we will contact the corresponding authors through E-mail. The information extracted from published articles will also be compared with the information from *ClinicalTrial.gov* registration platform if available, any significant inconsistency will be considered to be “high risk of bias” and recorded.

### Data synthesis

First of all, we will summarize the study design and demographic feature of each included study as table and in text. Specifically, information including the year of publication, sample size, drug usage, treatment course, outcome measurements, etc. of each individual study will be presented as a summary table. The results of each kind of drugs based on the same hypothesis will be described in the text.

### Quantitative synthesis

**Fig. 2:**
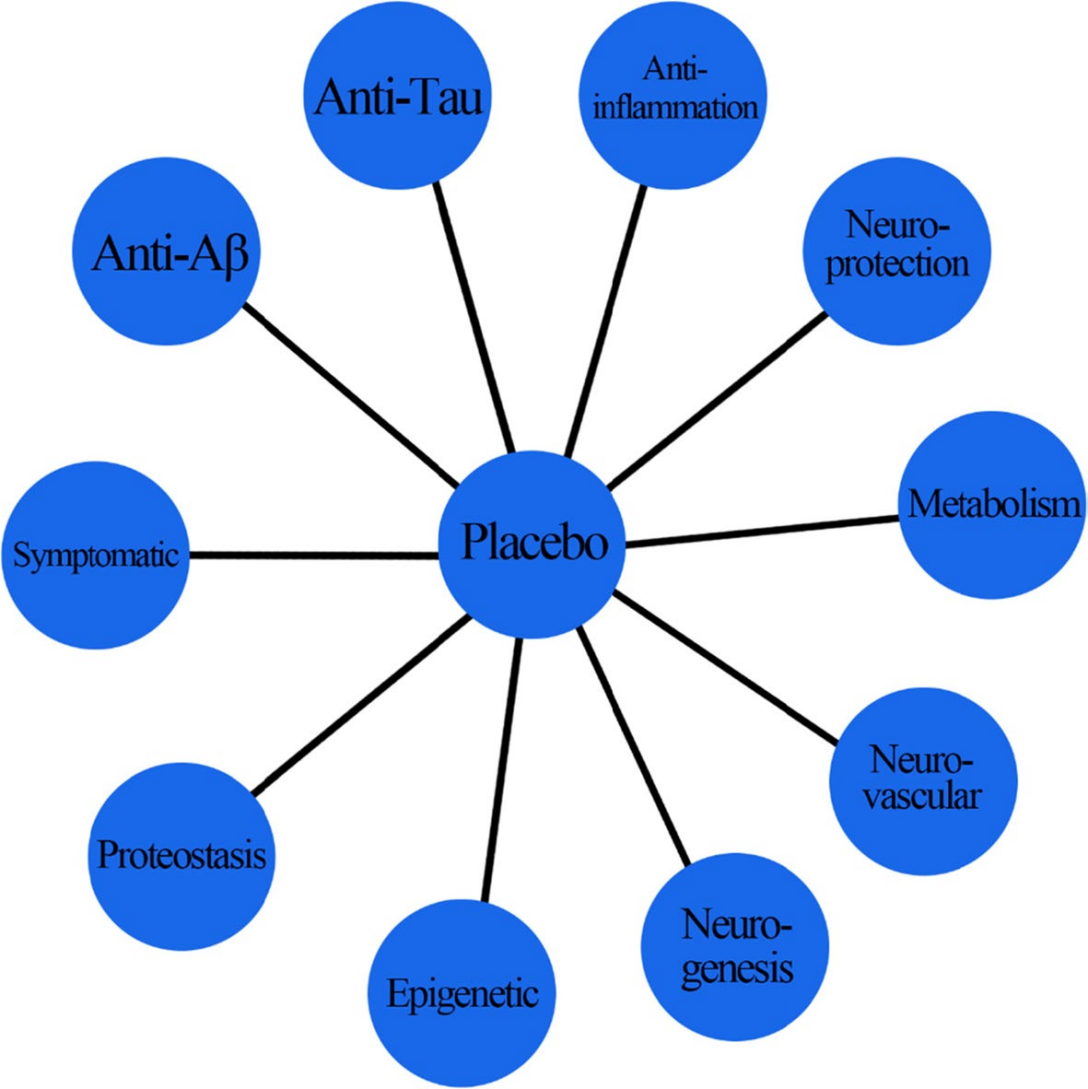
Schematic of indirect treatment comparisons between different kinds of drugs within the network meta-analysis framework

In phase III clinical trials, scientists usually compare experimental drugs with placebo rather than other experimental drugs. Thus, there may be a lack of head-to-head comparisons. To achieve reliable quality on indirect treatment comparisons, we will evaluate the study design, demographic feature, stage of AD, and usage of different kinds of drugs to identify potential confounding factors and test the homogeneity and similarity. Any studies with significant heterogeneity will be excluded from the network meta-analysis. By setting placebo groups as the referent group, we will generate a network with all included studies as Fig. 2.

To perform pooled estimations, R 4.0.5 software (http://www.r-project.org) with “gemtc” package (http://cran.r-project.org/package=gemtc) and JAGS 4.3.0 software [39] will be used to make a Bayesian network meta-analysis. We will perform analyses using random-effect model to accommodate potential heterogeneity. We will perform convergence diagnostics through “gelman.plot” command to test the convergence of the models. Trace plot and density plot will be applied to determine the best parameters of the number of adapt iterations, the number of iterations, and thin. For primary outcomes, we will present pairwise comparisons within the networks as tables and forest plots. Probability bars of ranking likelihood estimations will be presented for each treatment in the networks.

### Assessment of publication bias

Although most of the published studies suggested negative results, we will assess the potential publication bias by performing funnel plot. We will examine the shape of the funnel plots to determine the existence of publication bias. If the shape seems asymmetrical, we will carefully discuss about potential publication bias and whether it will affect our results.

### Subgroup analysis

We will perform subgroup analysis on different stages of AD. Potential subgroup analysis will also include the administration of the drugs (oral, muscle injection or intravenous injection, etc.), the dosage of the drugs (high dosage or low dosage), the entire treatment course, and the duration of the follow-up period.

### Sensitivity analysis

The results of sensitivity analysis will also be reported for primary outcomes. We will perform sensitivity analysis for the pooled estimations by excluding original studies with high risk of bias. We will also perform sensitivity analysis by excluding original studies with relatively short follow-up duration.

### Grading of recommendations

We will use the Confidence In Network Meta-Analysis (CINeMa) tool [40] to evaluate the grade of recommendation for each comparison respectively. By uploading data to the website (https://cinema.ispm.unibe.ch/), we will assess the following domains for each outcome: within-study bias, reporting bias, indirectness, imprecision, heterogeneity, and incoherence [40]. For each domain, we will give an evaluation of “no concerns,” “some concerns,” or “suspected” and finally give a confidence rating as “very low,” “low,” “moderate,” or “high” for each individual comparison.

## Discussion

To our knowledge, this will be the first systematic review and network meta-analysis that qualitatively and quantitatively analyze the existing clinical trials of anti-AD drugs. Through comparing the cognitive benefits of drugs based on different hypothesis, this study may reveal the key factor of treating AD and protect cognitive function. This Bayesian network meta-analysis will also rank the effects of different hypotheses on improving cognitive function and treating AD. Our results may be helpful for further drug development and clinical practice.

However, there are some limitations of our study. We will only search the literature databases in English. Although we will not limit the language of the literature, and studies with high quality were usually published in English or included in English databases, this may lead us to miss some important studies. In addition, we will only search the *ClinicalTrial.gov* registration platform, and the other registration platforms will not be searched. To avoid potential missing, we will search the AlzForum website as a supplement, but it may still miss some newly developed drugs and new researches.

## Supplementary Information

**Additional file 1.** PRISMA-P 2015 Checklist.

**Additional file 2.** Search strategy.

## Data Availability

Data sharing is not applicable. We will report all data in the systematic review and network meta-analysis.

## Abbreviations

Aβ: Amyloid-β
AD: Alzheimer’s disease
ADAS-Cog: Alzheimer’s Disease Assessment Scale-Cognitive Section
ADL: Activities of daily living
ATN: β amyloid deposition, pathologic tau, and neurodegeneration
CDR-SOB: Clinical Dementia Rating-Sum of Boxes
CINeMa: Confidence In Network Meta-Analysis
IWG: International Working Group
MMSE: Mini-Mental State Examination
NINCDS-ADRDA: National Institute of Neurological and Communicative Disorders and Stroke-Alzheimer’s Disease and Related Disorders Association
NMA: Network meta-analysis
NMDA: *N*-methyl-*D*-aspartic acid
PRISMA: Preferred Reporting Items for Systematic Review and Meta-Analysis
PRISMA-P: Preferred Reporting Items for Systematic Review and Meta-Analysis-Protocols
PROSPERO: International Prospective Register of Systematic Reviews
RCT: Randomized controlled trial
RoB2: Risk of Bias tool 2
SD: Standard deviation
YLL: Years of life lost
