# Supplementary material for: Effects of different kinds of anti-Alzheimer’s disease drugs on cognitive improvement: protocol for a systematic review and network meta-analysis of phase III clinical trials
Source: Syst Rev. 2022 May 3;11:84. doi: 10.1186/s13643-022-01964-x (PMC9063050; doi:10.1186/s13643-022-01964-x)
Supplement: Supplementary file 2 — Additional file 2. Search strategy. [file 13643_2022_1964_MOESM2_ESM.pdf]

Search strategy for PubMed-Ovid:

Search	Query
#1	exp Alzheimer disease/ or dementia/
#2	(Alzheimer* or dement*).tw.
#3	(cognit* or memor*).tw.
#4	(impair* or decline or reduc*).tw.
#5	3 and 4
#6	1 or 2 or 5
#7	exp randomized controlled trials/
#8	(clinical and trial).mp.
#9	exp clinical trial/ or random*.mp. or drug therapy.mp.
#10	‘randomized clinical trial’.mp. or ‘controlled clinical trial’.mp. or ‘placebo’.mp. or ‘drug therapy’.mp. or random*.mp.
#11	7 or 8 or 9 or 10 or 10
#12	‘phase III’.mp. or ‘phase 3’.mp. or ‘phase III’.mp.
#13	6 and 11 and 12

Search strategy for EMBase-Ovid:

Search	Query
#1	exp Alzheimer disease/ or dementia/
#2	(Alzheimer* or dement*).tw.
#3	(cognit* or memor*).tw.
#4	(impair* or decline or reduc*).tw.
#5	3 and 4
#6	1 or 2 or 5
#7	exp randomized controlled trials/
#8	(clinical and trial).mp.
#9	exp clinical trial/ or random*.mp. or drug therapy.mp.
#10	‘randomized clinical trial’.mp. or ‘controlled clinical trial’.mp. or ‘placebo’.mp. or ‘drug therapy’.mp. or random*.mp.
#11	7 or 8 or 9 or 10 or 10
#12	‘phase III’.mp. or ‘phase 3’.mp. or ‘phase III’.mp.
#13	6 and 11 and 12

Search strategy for Cochrane CENTRAL:

Search	Query
#1	MeSH descriptor: [Alzheimer Disease] explode all trees
#2	‘Alzheimer*’:ti,ab,kw OR ‘dement*’:ti,ab,kw
#3	‘cognit*’:ti,ab,kw OR ‘memor*’:ti,ab,kw
#4	‘impair*’:ti,ab,kw OR ‘decline’:ti,ab,kw OR ‘reduc*’:ti,ab,kw
#5	#3 AND #4
#6	#1 OR #2 OR #5
#7	randomized controlled trial:pt or clinical trial:pt

#8	('clinical':ti,ab,kw AND 'trial':ti,ab,kw) OR 'clinical trial':ti,ab,kw OR random* OR 'drug therapy':ti,ab,kw
#9	#7 OR #8
#10	‘phase III’:ti,ab,kw OR ‘phase 3’:ti,ab,kw OR ‘phase III’:ti,ab,kw
#11	#6 AND #9 AND #10

Search strategy for *ClinicalTrials.gov*:
Status: All studies
Condition or disease: Alzheimer disease
Study type: Interventional Studies (Clinical Trials)
Phase: Phase 3

Search strategy for Google Scholar:
(Alzheimer* OR dement*) AND (randomized controlled trial) AND (phase 3)

Search strategy for AlzForum:
FDA STATUS: Phase 2/3 AND Phase 3
CONDITION: Alzheimer’s disease
